# Critical Components for Participation and Personal Recovery in the Flexible Assertive Community Treatment (FACT) Model: A Case Study of the Delivery Process

**DOI:** 10.5334/ijic.9814

**Published:** 2026-03-06

**Authors:** Madeleine Borgh, Ulrika Bejerholm, Elisabeth Argentzell, Sonya Girdler, Annika Lexén

**Affiliations:** 1 Faculty of Medicine, Department of Health Sciences, Lund University, Lund, Sweden; 2 Department of Psychiatry, Region Skåne University Hospital, Lund, Sweden; 3 Curtin Autism Research Group, School of Allied Health, Curtin University, Perth, Western Australia, Australia; 4 Karolinska Institutet for Neurodevelopmental Disorders (KIND), Department of Women’s and Children’s Health, Karolinska Institutet, Stockholm, Sweden

**Keywords:** complex healthcare interventions, integrated mental health services, mental health recovery

## Abstract

**Introduction:**

Flexible Assertive Community Treatment (FACT) is implemented in Swedish general Mental Health Services. While FACT focuses on supporting the participation and personal recovery of individuals with complex mental health needs, critical components enabling these aspects are poorly understood. The aim of this study was to understand the critical components of FACT delivery supporting participation in everyday life and personal recovery in *general* Mental Health Services, and to understand how these critical components relate to the focus areas in FACT.

**Methods:**

A single-case study design with two embedded units of analysis was conducted. Data was collected through two focus groups, ten fidelity assessments and eight interviews between 2020 to 2024. The data was analysed using qualitative content analysis and pattern matching.

**Results:**

Critical components were related to *Placing service users at the centre through an integrated multiprofessional team, Targeting service users’ needs through continuity and flexibility* and *Empowering service users in their everyday life*. Most critical components included several focus areas in FACT.

**Conclusion:**

An integrated multiprofessional team providing individualised and flexible care and support can support service users in participating in everyday life and promoting their personal recovery.

## Background

While Mental Health Services (MHS) in Sweden have historically been underpinned by bio-medical perspectives, parallelling developments in other countries, there has been a national shift towards more recovery-oriented approaches [1]. This change has driven a need for research evaluating new approaches to providing mental health care and support [2], particularly approaches aiming to support individuals with complex mental health needs. This refers to individuals with at least one psychiatric diagnosis with fluctuating mental health with difficulties participating in the community [3,4]. In Sweden the challenges experienced by this group mean they require support from *general* MHS (for care and treatment) and Social Services (providing support and rehabilitation) which largely operate independently under different regulations [5,6].

Internationally, and in Sweden, research into recovery-oriented services (ROS) is a priority [1,7]. One ROS implemented in European countries [8–13] and now adopted in Sweden [14] is the Flexible Assertive Community Treatment (FACT) model. FACT is an outpatient service targeting recovery through eight focus areas: *(1) flexible care, (2) personal domain, (3) participation domain, (4) symptomatic domain, (5) planning and monitoring, (6) crisis and safety, (7) network collaboration*, and *(8) quality and innovation*. The flexibility inherent in FACT is assumed to support the delivery of tailored and individualised interventions [15,16] providing care and support responsive to individual’s needs and goals [3]. Previous research has highlighted its impact on individuals in improving psychosocial functioning [14] and reducing symptoms [17]. Additionally, FACT has been shown to improve patient compliance [8], increase outpatient contacts [9,14] and reduce hospital admissions [9,12,13]. While there is emerging understanding of the impact of FACT on personal recovery and participation in everyday life, further research is needed in identifying and clarifying the critical components driving these outcomes informing ongoing model development [3]. Critical components in models of service are comprised of a combination of resources and human reasoning, commonly referred to as mechanisms [18].

The present study is grounded in the underlying principle that participating in everyday life promotes personal recovery [3] with participating in meaningful activities playing a crucial role in this process [19]. Recovery is commonly understood as clinical and personal recovery [20]. While clinical recovery focuses on symptom reduction through medication and treatment, personal recovery views an individual’s life situation holistically [1] promoting self-agency and control over one’s life despite the presence of ongoing psychiatric symptomatology [21]. The CHIME framework conceptualises personal recovery as emerging from five key processes: *connectedness, hope and optimism, identity, meaning and purpose*, and *empowerment* [22]. The FACT model integrates these processes by adopting a respectful and affirmative approach that emphasise service users’ strengths [15,16] and facilitate their participation in everyday life [3].

Participation is defined in the International Classification of Functioning, Disability and Health (ICF) as “involvement in a life situation” [23, p.10]. It encompasses various meaningful and valued life domains essential for mental wellbeing [23]. FACT recognises that participating in major life areas such as self-care, work, education, leisure and social networks is critical to personal recovery [15,16].

Our previous research on the FACT model within *general* MHS in Sweden, has focused on examining service users’ experiences [3]. This work has highlighted genuine relationships between service users and team members in delivering flexible and integrated care characterised by collaboration and recognition of the individual in supporting their participation in everyday life and the personal recovery process. Examining the critical components of FACT relating to participation in everyday life and personal recovery is the next important step in developing this model. Therefore, one important aspect is understanding FACT from a service delivery perspective. Previous research has highlighted aspects that may be crucial for understanding these components and how they support service users’ participation in everyday life and personal recovery [3,24–26]. A systematic review identified components important to ROS in facilitating personal recovery, including establishing relationships between service users and professionals, empowering service users, and using peer supporters as role models [27]. In youth FACT, a multidisciplinary, integrated, and collaborative approach, combined with flexible care and support was central [24]. Similarly, our previous study [3] highlights participating in meaningful activities as a key factor in promoting recovery. Assertive Community Treatment (ACT) further emphasises team-member engagement, relationship building [25], and autonomy [26] in the recovery process. Based on insights from previous research, the FACT model, and the theoretical foundations of participating in everyday life and personal recovery, we can hypothesise potential critical components of FACT. Among these, flexibility and collaboration with networks, alongside participatory and personal domains, appear central. However, further research is needed to better understand these components of the FACT model. Such an in-depth understanding would complement quantitative findings [28], strengthening the evidence for further development of the model. Therefore, the aim of this study was to understand the critical components of FACT delivery supporting participation in everyday life and personal recovery in *general* MHS, and to understand how these critical components relate to the focus areas in FACT.

## Method

### Study design

An explanatory single-case study with embedded units of analysis (hereafter referred to as units) was applied, given its utility in studying real-world phenomenon within a specific context [29]. The study design was grounded in the theoretical notion that participation in everyday life can support personal recovery, a stance underpinning the study propositions (supporting and rival) outlined in the study protocol (Table 1). This aligns with the authors theoretical expertise as occupational therapists. Furthermore, all authors have lived experience as professionals, by working with and conducting research on individuals with complex mental health needs and integrated care and support. MB has experience of mental illness and being a service user in MHS, while UB and AL have experiences as relatives. In addition, all authors except MB have extensive knowledge in implementation of complex interventions targeting MHS and/or individuals with complex mental health needs.

**Table 1:** Study propositions based on theoretical underpinnings that participation can support personal recovery in Flexible Assertive Community Treatment.


*Supportive*	1. The FACT model contributes positively to participants’ participation in everyday life and personal recovery.

	2. Team members building relationships with service users enable participants’ participation in everyday life and personal recovery.

	3. Focus areas 1–3, and 7 are most important in supporting participation in everyday life and personal recovery.

*Rival*	4. In addition to FACT, service users receive further care and support contributing to their participation in everyday life and personal recovery.

	5. Participants’ personal circumstances changed, increasing participation in everyday life and personal recovery.


The study was conducted as part of a randomised controlled trial (RCT) (FORTE Dnr 2019-00073) of FACT within *general* MHS in Southern Sweden. The aim of the RCT is to examine the effectiveness of FACT on service users’ health and everyday functioning (primary outcomes), as well as personal and clinical recovery and healthcare and social service use (secondary outcomes), compared to treatment as usual among individuals with complex mental health needs.

### FACT

FACT is described in accordance with the Template for Intervention Description and Replication (TIDieR) checklist [30]. FACT is a development of ACT [31], designed to support individuals with complex mental health needs [4]. Care and support are focused around the eight focus areas, ensuring interventions are aligned with national guidelines and recovery-oriented practice [15,16]. FACT focuses on integrating Mental Health and Social Services, involving professionals from different disciplines, collaborating in providing scalable and tailored outpatient care and support in the community. The multiprofessional team should include occupational therapists, peer supporters, employment specialists, social workers, nurses, psychologists, psychiatrists and coordinators from Social Services [15]. Team members are responsible for up to 15 service users [15,16], coordinating care and support and drawing on expertise within the broader team as needed. During times of crisis, care and support are provided according to ACT principles, where the whole team shares responsibility for service users. This is facilitated through daily FACT meetings with service users listed on a FACT board for continuous follow-up as well as unscheduled time (‘white days’), allowing for assertive outreach and collaboration with formal and informal networks [15,16]. Collaboration is further supported by using Coordinated Individual Plans (CIP), a legally regulated written plan ensuring collaboration and integrated care and support [5].

### Case and setting

The case was defined as the process of providing care and support to service users within a FACT team in the context of *general* MHS in southern Sweden, working collaboratively with Social Services. This case consists of two FACT teams (units) operating within the same health region and participating in the larger RCT. The FACT teams achieved an average program fidelity score of 8.8 and 10.1 respectively (out of a maximum score of 13). In Team 1, there was no psychologist until 2022, and no full-time occupational therapist or peer support worker until 2024. Team 1 also lacked an employment specialist during the study period. Related to expertise, the team lacked substance use expertise in 2020 and 2023. Throughout the study period, Team 2 had professionals fulfilling all roles specified by the FACT model but was without a substance use expert in 2023. FACT Team 1 covered eight municipalities in southern Sweden, while FACT Team 2 covered ten. Care and support were provided to individuals with complex mental health needs, including psychiatric diagnoses such as depression, attention deficit disorders, personality disorder, bipolar disorder, post-traumatic stress disorder (PTSD), and substance use problems. However, individuals with psychosis and substance use problems as their primary diagnosis were excluded, as these service users were referred to other specialist MHS.

### Participants and data collection

Data was collected from 2020 to 2024, through focus group interviews with team members, program fidelity assessments, and pairs/triadic interviews with team members. All team members from the two FACT teams were eligible to participate in the focus groups and interviews. The team leader (team 1) and the head of unit (team 2) were invited and informed about the aim of the focus groups and interviews. They subsequently informed the team members of the opportunity to participate, with the first author (MB) contacting team members via e-mail for the interviews.

Focus groups and interviews were conducted with representatives from all professional roles, including one head of unit (Table 2), thereby capturing a range of different perspectives. However, psychologists were absent on the day of the focus groups and, therefore, did not participate. Four team members took part in both the focus groups and interviews. Informed consent was obtained prior to data collection and highlighted the voluntary nature of participating and the opportunity of withdrawal. The study was approved by the Swedish Ethical Review Authority (Dnr 2019-02866/2022-05512-02).

**Table 2:** Detailed description of data sources used in a case study of participation and personal recovery in Flexible Assertive Community Treatment.


DATA SOURCES	DESCRIPTION

Focus groups (n = 2)	One focus group with FACT team 1 and FACT team 2 respectively (2020). Focus group 1: Psychiatrist, two social workers, two carers, medical secretary, head of unit, two Social Service coordinators. Focus group 2: Psychiatrist, nurse, social worker, occupational therapist, peer supporter, work specialist, four Social Service coordinators.

FACT fidelity assessment (n = 10)	Five FACT fidelity assessments for FACT team 1 and FACT team 2 respectively (2020-2024), including fidelity reports and team documents.

Interviews (n = 8)	Eight interviews with FACT team members from both teams in pairs and triads (2024). Interview 1.1: Two Social Service coordinators Interview 1.2: Nurse, two carers Interview 1.3: Psychologist, social worker Interview 1.4: Psychiatrist, nurse, medical secretary Interview 1.5: Two occupational therapists, social worker Interview 2.1: Two Social Service coordinators Interview 2.2: Psychologist, nurse Interview 2.3: Work specialist, peer supporter


#### Focus groups

Two focus groups were conducted by the last author (AL) together with another researcher as part of a process evaluation within the larger project. A semi-structured interview guide underpinned by process evaluation theory [32] was developed by UB in collaboration with AL. It covered topics such as fidelity, dose, reach, recruitment, and context. Examples of questions included are: *“On a scale of 1–10, to what extent do you think it has been possible to organise care according to FACT in your organisation?”* and “*Do you feel that the different FACT focus areas have been achieved/implemented as expected?”*

#### Fidelity assessments

Ten FACT fidelity assessments, conducted by two external auditors on an annual basis and aligned with the most recent Swedish version of the FACT model, contributed to the data [15]. During four fidelity assessments, the external auditors consulted with AL (as a FACT expert) when deemed necessary. The fidelity assessment was structured around two sections. In Section A, quantitative information was collected, such as team members and structure. In section B, qualitative data was gathered and summarised about the eight focus areas as reported by team members, service users, significant others, and described in team documents. More detailed description can be found elsewhere [15].

#### Interviews

Interviews were conducted by the first author (MB). A semi-structured interview guide was created in accordance with the study aim and theoretical underpinnings. Examples of questions included: *“What do you think helps you to support participants’ personal recovery process?”* Additional questions explored how team members supported service users’ participation in the activity areas of *self-care, domestic life, interpersonal interactions and relationships, major life areas* and *community, social and civic life*, as defined by the ICF [23]. A pilot interview was conducted and included in the present study, given no changes were made to the interview guide.

### Data analysis

**Figure 1:**
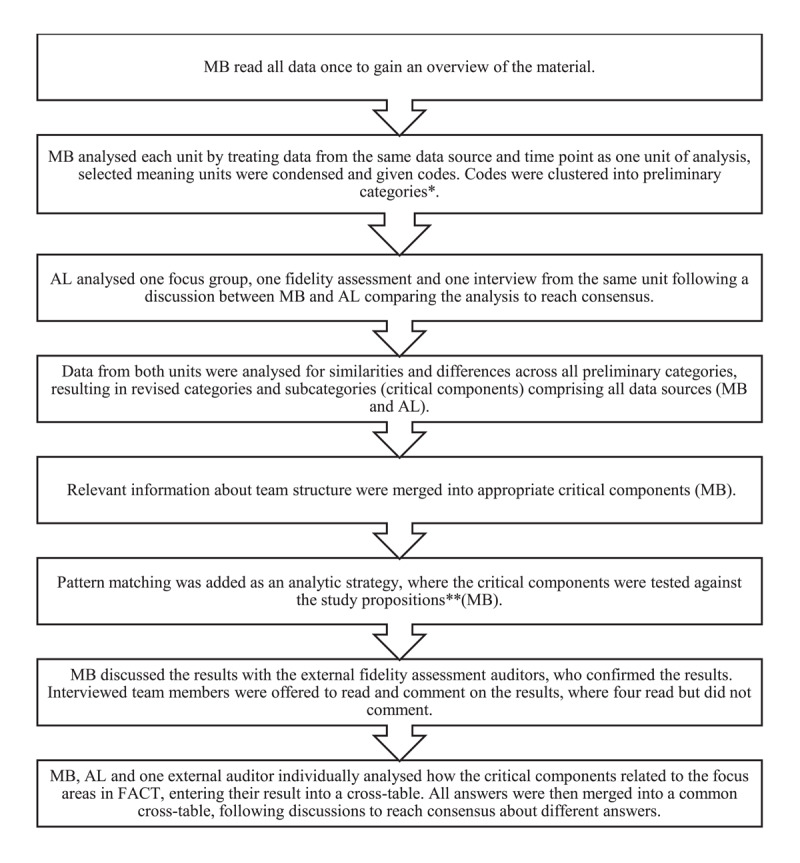
The analytical process used in a case study of participation and personal recovery in Flexible Assertive Community Treatment. *[34,35]. **[29].

All focus groups and interviews were audio-recorded and transcribed verbatim. All data sources were then imported into NVivo 14 [33], serving as a database for the analysis. Data were analysed using qualitative content analysis [34], applying an unconstrained deductive approach [35] guided by the study’s theoretical underpinnings, as well as by applying pattern matching [29]. Additionally, results were analysed in accordance with the focus areas in FACT. Throughout the analytical process, the authors met regularly to reach consensus. When disagreement arose, the authors was guided by the study aim to find appropriate focus. See Figure 1 for a detailed description of the analytical process.

## Results

The results are presented in two parts. The first, *Flexible and continuous support by a multiprofessional team enables participation in everyday life as part of the personal recovery process* describes the critical components of the FACT delivery. The second part, *Critical components and FACT focus areas*, presents the alignment between the critical components and each of the focus areas in FACT.

### Flexible and continuous support by a multiprofessional team enables participation in everyday life as part of the personal recovery process

**Figure 2:**
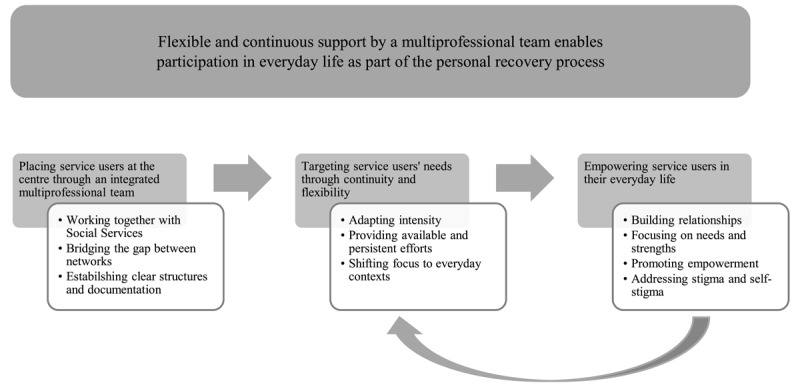
Critical components supporting participation and personal recovery in Flexible Assertive Community Treatment, from a service delivery perspective.

Figure 2 illustrates how critical components shape the care and support process that foster participation in everyday life as a part of the personal recovery process. The categories identified were *Placing service users at the centre through an integrated multiprofessional team, Targeting service users’ needs through continuity and flexibility*, and *Empowering service users in their everyday life*. Each category encompassed several critical components, described below. All data sources are represented in each critical component, and indicated as follows: focus groups^A^, fidelity assessments^B^, interviews^C^.

#### Placing service users at the centre through an integrated multiprofessional team

This category consists of three critical components: *Working together with Social Services, Bridging the gap between networks*, and *Establishing clear structures and documentation*. These components emphasise the team’s collaboration and structured approach to focusing on participants’ needs.

##### Working together with Social Services

The multiprofessional team, taking a team approach ^A,B,C^ allowing for adequate time and personnel resources, was the foundation for organising care and support^A,C^. Social Services coordinators were considered formal members of the FACT team, providing a bridge between Mental Health and Social Services^A,B,C^, promoting efficiency and mutual understanding^A,C^. As one participant described, “*It’s not like […] the municipality and the region are over there, we are together, and both parties are important*”^A^. While the approaches to collaborating with and communicating between Mental Health and Social Services within each team varied, with Team 2 having a process leader from the beginning and Team 1 receiving this support two years later, these processes were continually evolving^A,B,C^. Barriers to collaborating and communicating included various legal obligations and organisational structures, staff turnover, unclear responsibilities between services^A,C^, interpreting service users consent for collaboration^C^, variations between different Social Services support^A,C^, and barriers to sharing information across municipal departments^C^. Early in the implementation of FACT, balancing the contact person role with professional obligations was reported as difficult^A^, and concerns about maintaining flexibility with larger caseloads were described^A,C^.

##### The gap between networks

Given their overarching responsibility for service users, the FACT team “*…establish[ed] collaboration with everyone around”*^A^, drawing on formal and informal networks^A,B,C^. Collaborations were established over time, through proactive network mapping, and fine-tuning ways of collaborating^B,C^, including offering CIP meetings^B,C^. Depending on their current ‘case-mix’ the FACT team collaborated with various formal services^B,C^, with contact initiated by the team or service user^A,B,C^. For example, team-level collaboration included partnerships with other Mental Health Services and providing professional supervision to Housing Assistance workers supporting individuals with psychiatric disabilities residing in the community, family homes and treatment centres^A,B,C^. Social Service coordinators were key in linking with municipal networks^A,B,C^. An example of service user level collaboration, was actively supporting service users in seeking information, assisting them before and in meetings with other services^C^ including Primary Health Care^A,B,C^. Challenges included staff turnover and communication barriers^A^. One team member noted limited opportunities to support service users in accessing civic society networks^C^.

Collaborating with service users’ informal networks (close relations) was emphasised as important in ensuring naturalistic supports^B,C^. With service users’ consent, key individuals attended meetings and were actively involved in interventions^A,B,C^ . While teams offered family training or counselling, they expressed a wish to extend this opportunity more broadly to others within service users networks^C^.

##### Establishing clear structures and documentation

Structure was essential in ensuring appropriate care- and crisis plans^B,C^, team documents, and FACT meetings. Care plans were detailed, outlining individualised goals, interventions and responsibilities^B^ with team members collaborating with service users in formulating, evaluating and revising care plans as needed^B,C^. One team was encouraged to more clearly document the care process^B^, and was reminded that “*the care plan is based on the participant’s goals and does not focus on problems*”^B^, with long-term participatory and personal recovery goals being the focus^B,C^. Care- and crisis plans were made accessible to other Mental Health Care units^B^. CIP informed planning and documentation together with coordinators from Social Services^A,B^, as coordinators lacked access to the Mental Health record system.

As these plans were developed collaboratively, with both service users and team members, team documents included team instructions and progress and were updated as procedures evolved. For example, systematic evaluation was identified as necessary, leading to the implementation of Routine Outcome Monitoring tools^B^. Daily FACT meetings often occurred^B^, and FACT boards were an important tool for planning and organising care and support^A,B^. While FACT meetings provided structure and efficiency^B,C^, they were at times contributing to stress^C^. Additionally, team members communicated daily, though some expressed a need for more dedicated team time^C^.

#### Targeting service users’ needs through continuity and flexibility

This category consists of three critical components: *Adapting intensity, Providing available and persistent efforts*, and *Shifting focus to everyday contexts*. These components were facilitated by the teams’ integrated and structured collaboration with surrounding networks.

##### Adapting intensity

Adapting the level of care and support was key in FACT^A,C^,, where both teams had consistently implemented flexible care^B^. This approach evolved over time with both teams refining routines, enabling organisational flexibility. Team 1 experimented with their approach, trialing both ‘white days’ or flexible schedules as approaches to supporting service users^A,B,C^. As one team described: “*So you don’t usually have a completely packed schedule, there is room for extra visits, or you adjust based on patients’ needs*”^C^. To maximise benefits from the ACT approach and benefit from ‘white days’, service users needed to engage with multiple team members^B^.

Decisions regarding changing the intensity of support were often made in collaboration with service users^A^ and were seen as a team responsibility^B^. Some team members stressed avoiding rapid upscaling^C^, while emphasising transparency with service users and their families^A^. Social Service coordinators played a key role in detecting needs early^A,B,C^, although some coordinators felt their ability to adjust the intensity of support as limited due to municipal regulations and their exclusions from ‘white days’^C^. While downscaling care and support was described as important, it was challenging^A,B,C^.

##### Providing available and persistent efforts

Developing responsive and consistent strategies towards service users was an ongoing process^C^, described as critical for establishing continuity in support^A,C^, and crisis prevention^C^. This comprised several actions by team members, including being available towards service users^A,C^ and repeatedly motivating service users to engage in interventions^B,C^. This had the potential of reducing the number of times service users contacted team members^A,C^ and crises events^A^. Service users were encouraged not to cancel appointments with the FACT team^B^, enhancing compliance. In the event where a service user required inpatient care, team members “…*conduct[ed] outreach and offer[ed] collaboration and support*”^B^, allowing for immediate follow-up and support ^A,B^.

##### Shifting focus to everyday contexts

Meeting service users in their everyday contexts facilitated understanding of their needs and experiences^A,B,C^, with teams consistently reaching out to service users in their communities^B^. Team members preferred to organise meetings in naturalistic settings such as in service users’ homes, going for a walk^A,B,C^, visiting the gym^A,C^, having coffee at a café^B,C^ or accompanying service users to other Health Care Services^A,C^. With consent from the service users, care plan or CIP meetings^B^, meetings with psychiatrists^A^, and occupational therapy interventions^C^ frequently occurred in service users’ homes. The location of meetings often aligned with their purpose, for example accompanying service users to other Health Care units was a step towards service users being able to attend their appointments independently^C^. As one team member described, “*a large part of the visits should be outside our premises […] because life is really not going on here [FACT clinic]…”*^C^. The FACT teams actively supported service users to remain in their homes during times of crisis, rather than admission to inpatient care^B,C^ requiring team members to take positive risks^B,C^.

#### Empowering service users in their everyday life

This category consists of four critical components: *Building relationships, Focusing on needs and strengths, Promoting empowerment*, and *Addressing stigma and self-stigma*. The flexible and continuous care and support delivered by the FACT team facilitated relationships that enabled the other components.

##### Building relationships

Building relationships with the service users from the onset of care and support was essential in encouraging service users to participate in their care and support process^A,B,C^,, described as “*…the very basis of the work, an alliance […] because if you don’t have that, then you have nothing to work with*”^C^. A foundation of openness, trust, and mutual respect was crucial when building relationships^A,B,C^. Each relationship was unique, requiring team members to adapt their interaction style to each service user^C^. These relationships served as a springboard for service users to explore other social contexts and discuss sensitive topics such as suicidal thoughts, personal care, housing, work, and relationships. For example, one team member emphasised the value of sharing experiences with service users to normalize feelings and behaviours^C^.

##### Focusing on needs and strengths

Seeing the whole individual while emphasising needs and strengths was central^A,B,C^, expressed as *“I think mainstream psychiatry is far too much about diagnoses, far too little about people, what they want and desire…”*^C^. Team 2 made efforts to match service users with a contact person based on shared interests^B^. While a strengths-based approach was seen as contributing to longer intervention periods, with the team responding to users fluctuating needs^A,B,C^, this approach was often challenging to maintain during periods of crisis. Several team members felt that service delivery in *general* MHS still largely relied on a bio-medical, rather than a recovery-oriented perspective^C^.

The FACT team continuously worked with service users in exploring what brought meaning to their lives^B,C^. While service users valued participating in social contexts^A,B,C^, noted barriers included a lack of social opportunities and sufficient access to peer supporters^C^. During times of crisis team members supported service users in identifying social contexts or problem-solving ways of continuing to participate in their interests^B,C^. Focusing on strengths, some FACT team members highlighted service users’ accomplishments related to their everyday lives^B,C^. Several team members supported service users in participating in major life areas, and social and civic life^A,B,C^, such as engaging in studies or clubhouse activities^C^.

##### Promoting empowerment

Team members sought opportunities to empower service users who were fundamentally viewed as experts in managing their lives^B,C^. “*Try to sit on your hands for a while to listen […]*^C^”, was an approach helping to give service users a sense of ownership and taking responsibility over their care and support processes^A,B,C^. While team members offered various interventions available, acting as advisers and facilitators^A,C^, ultimately service users choose whether to accept these interventions or not^A,C^. Team members noted that given the involuntary nature of compulsory inpatient care service users self-determination was constrained during these admissions^B,C^. While team members sought not to be too directive when working with service users^A,C^, one team member perceived the current approach to delivering care and support as too paternalistic, advocating for more dialogue with the service users across all stages of the process^C^. While strategies such as brief admission^B,C^ and second opinions from other health professionals were used in supporting service users to regain control over their lives^B^, team members highlighted that at times they needed to advocate on behalf of service users^B,C^.

##### Addressing stigma and self-stigma

Stigma and self-stigma were tackled through two approaches: a non-judgmental team culture, and directly supporting service users^A,B,C^. The team process was underpinned by a vision of “*always meet[ing] our service users and each other with a non-judgmental approach*”^B^. Strategies employed in working towards this vision included having ethical rounds^B,C^, discussing dilemmas relating to stigma and self-stigma, and reminding team members of appropriate language use^B^. Peer supporters played a key role in efforts aiming to reduce stigma^A,B^. Approaches directly addressing the issue of stigma and self-stigma included Narrative Enhancement – Cognitive Therapy (NECT)^B^ and engaging with peer support workers^A,B^. Here, one team member addressed the need for more peer support workers^C^. Addressing hope, acceptance, normalising feelings and behaviors, and letting go of one’s patient identity was also important^A,B,C^. Interestingly, one team member noted that the MHS itself contributed to service users internalising the sick role^C^.

In summary, critical components of the FACT model were centred around a multiprofessional and integrated team applying a flexible and continuous approach. This facilitated relationships supporting service users in building on their strengths in everyday life, while also addressing empowerment, and stigma.

### Critical components and FACT focus areas

The majority of the critical components comprised two to five different focus areas (see Table 3). However, *Building relationships* and *Addressing stigma and self-stigma* included only one focus area. Together, this suggests that the focus areas are generally implemented as an integrated whole.

**Table 3:** Critical components related to participation and personal recovery in Flexible Assertive Community Treatment in relation to its focus areas.


	1. FLEXIBLE CARE	2. PERSONAL DOMAIN	3. PARTICIPATION DOMAIN	4. SYMPTOMATIC DOMAIN	5. PLANNING AND MONITORING	6. CRISIS AND SAFETY	7. NETWORK COLLABORATION	8. QUALITY AND INNOVATION

**Placing service users at the centre through an integrated multiprofessional team**								

Working together with Social Services	X				X		X	

Bridging the gap between networks	X		X		X		X	X

Establishing clear structures and documentation	X		X		X	X		X

**Targeting service users’ needs through continuity and flexibility**								

Adapting intensity	X			X		X		

Providing available and persistent efforts	X		X			X	X	

Shifting focus to everyday contexts	X	X						

**Empowering service users in their everyday life**								

Building relationships		X						

Focusing on needs and strengths		X	X					

Promoting empowerment		X	X		X			X

Addressing stigma and self-stigma		X						


## Discussion

This study advances the understanding of critical components within the FACT model that may support participation in everyday life and personal recovery among individuals with complex mental health needs, and how these components relate to focus areas in FACT. The results showed that *Placing service users at the centre through an integrated multiprofessional team* and *Targeting service users’ needs through continuity and flexibility* were important for *Empowering service users in their everyday life*. Furthermore, the results showed that most critical components included several focus areas in FACT [15,16]. Overall, these results support the notion that FACT can facilitate participation in everyday life and personal recovery, as suggested in Proposition 1 (Table 4). This is in line with previous studies about service users’ experiences of FACT [3,10]. However, implementing FACT also presented challenges. Below, the results will be discussed in relation to these challenges, study propositions, the FACT model, and previous research.

The categories *Placing service users at the centre through an integrated multiprofessional team* and *Targeting service users’ needs through continuity and flexibility* emphasised coordinated teamwork and across sector collaboration, while maintaining a structured approach to centring participants’ needs. Together, these critical components reflect a responsive form of service delivery central to ROS. The component, *Bridging the gap between networks*, indicated that collaboration at the team level primarily focused on connections within Mental Health and Social Services, whereas supporting service users’ participation in civic society remained challenging. Engagement with community networks mainly occurred at the individual level when supporting service users explore meaningful activities. Given that participation in meaningful social contexts is essential for personal recovery [19], strengthening formal collaboration with civic and community actors at the team level represents an important opportunity to advance recovery-orientation within FACT. Such collaboration would also reinforce links between citizenship [10], social inclusion, and recovery [36]. This highlights a wider need to develop and evaluate approaches that more systematically promote social inclusion, for example within Social Services, pointing to a key direction for future research and service development.

The critical component *Establishing clear structures and documentation*, although not included in the pre-defined propositions, underscored working systematically and goal-directed together with service users to stay focused on service users’ recovery, in line with the FACT model [15,16]. A notable challenge was that Social Service coordinators did not have access to the medical record systems used within MHS, an issue previously identified [11]. One way to bridge this gap was by using CIP. However, overreliance on verbal communication with Social Service coordinators could hinder service users’ personal recovery process, since Social Services play a crucial role in supporting service users’ participatory goals. In line with Trane et al [11], we argue that this is a matter of organisational readiness, and that both available structures and opportunities for sharing documentation must be carefully considered to ensure efficient integrated care and support. This represents an important area for further research within the Swedish context to further advance the FACT model.

The category *Empowering service users in their everyday life* highlighted how care and support was permeated by an individualised, recovery-oriented approach. Within this, the critical component *Building relationships* between service users and team members was key, confirming study Proposition 2 (Table 4), and aligning with previous studies of ROS [3,25,27]. These relationships were facilitated by organising care and support through a multiprofessional team, as well as the individualised flexibility and continuity inherent in FACT. However, such relationships are not explicitly emphasised in the FACT model [15,16]. As such relationships are closely linked to personal recovery [22] and person-centred care [37], explicitly incorporating relational aspects into FACT could further enhance the model and strengthen its capacity to deliver high quality, recovery-oriented care and support.

When relating the critical components to focus areas in FACT, most components incorporated several focus areas (Table 3). Thus, Study Proposition 3 (Table 4) was partially confirmed, as the focus areas *flexible care, personal domain, participation domain*, and *network collaboration* were corroborated as important for supporting service users. These results are in line with previous studies on FACT and ACT [24–26]. However, several critical components also included other focus areas, suggesting that multiple areas must be applied simultaneously to effectively support participation in everyday life and the personal recovery process. Attempts to isolate the impact of individual focus areas may therefore oversimplify the complexity of FACT. This finding is an important contribution for research regarding ROS and FACT, highlighting the importance of approaching service users holistically. Notably, the components *Building relationships* and *Addressing stigma and self-stigma* included only one focus area (Table 3). This contrasts to the results describing these critical components, which emphasise that these components are influencing many aspects of care and support. A potential future development of FACT could involve more clearly linking these components into multiple focus areas. For example, *Building relationships* was shown to be essential for supporting participation in social contexts, an aspect of the *Participation domain* in FACT.

In summary, this study identified critical components of the FACT model important for supporting service users’ participation in everyday life and in their personal recovery process, while also illuminating organisational barriers that can hinder recovery-oriented and integrated practice. Clinical implications based on these results are presented in Table 4. Together with our previous study [3], these results suggest that FACT is a ROS with the potential to deliver holistic and individualised care and support that addresses service users’ needs, where clinical and personal recovery perspectives can co-exist. Focusing on further developing and adapting FACT into Swedish context, and examining its effectiveness, are therefore important future research areas.

**Table 4:** Summary of results related to participation and personal recovery in Flexible Assertive Community Treatment and clinical implications.


PROPOSITIONS	RESULTS	CRITICAL COMPONENTS	CLINICAL IMPLICATIONS

*Supportive*			

1. The FACT model contributes positively to participants’ participation in everyday life and personal recovery.	Multiprofessional and integrated teams using a flexible and continuous approach was crucial. This facilitated relationships supporting service users in everyday life, while also addressing empowerment and stigma/self-stigma.	All	Care and support should be based on flexible, multiprofessional, and integrated teams.

2. Team members building relationships with service users enable participants’ participation in everyday life and personal recovery.	Building relationships was crucial, acting as springboards to social contexts and to discuss sensitive topics.	Building relationships	Develop strategies to build and maintain relationships with service users.

3. Focus areas 1-3, and 7 are most important in supporting participation in everyday life and personal recovery.	Consulted different professions within the team. Direct contact links to service users and other care and support. Adapted level of care and support together with service users. Viewing service users as experts in their lives, emphasising a team culture that recovery was possible. Directly supporting service users in addressing stigma/self-stigma. Focusing on long-term participatory and personal recovery goals. Supporting service users in sustaining/developing activities in everyday life, major life areas and social/civic life. Collaboration with formal and informal networks over time. Involving significant others or offering family training/counselling.	Working together with Social Services Bridging the gap between networks Adapting intensity Providing available and persistent efforts Focusing on needs and strengths Promoting empowerment Addressing stigma and self-stigma	Embrace multiprofessional perspectives and maintain direct contact links. Adopt a recovery-oriented culture at the team-level and prioritise building strong supportive relationships with service users. Together with service users, pinpoint activities in everyday life to include in care plans. Build strong networks on team-level, while supporting service users creating individuals’ networks.

*Rival*			

4. In addition to FACT, service users receive further care and support contributing to their participation in everyday life and personal recovery.	FACT teams are required to collaborate with formal and informal networks, where contact was established over time.	Bridging the gap between networks	Build strong networks based on service users’ needs.

5. Participants’ personal circumstances changed, increasing participation in everyday life and personal recovery.	Through genuine relationships, team members supported service users in sustaining/developing activities in everyday life, major life areas and social/civic life.	Building relationships Focusing on needs and strengths	Build relationships with service users and pinpoint activities in everyday life to include in care plans.


## Methodological considerations

The FACT model is a complex service, making a case study design an appropriate choice. However, the data for this case was drawn from only two FACT teams in Sweden by using a service delivery perspective, limiting the external validity of the results. This was addressed by grounding the study in theoretical underpinnings from previous research, allowing for analytic generalisations as described by Yin [29]. The use of pattern matching and propositions strengthened internal validity [29], with pattern matching leading to the confirmation or partly confirmation of supportive propositions. However, the rival propositions (Table 4) were not rejected, which could be seen as a limitation. Nevertheless, the analysis revealed that these aspects were already integrated into the FACT teams’ work, suggesting a broad understanding of factors important for participation in everyday life and personal recovery. The interview guide for the interviews was based on the theoretical underpinnings, but not all participation areas in the ICF were included. Yet, this was a deliberate strategy, to focus on participation areas that could directly relate to activities.

Regarding construct validity, multiple data sources were used and systematically analysed by applying investigator triangulation [29] and qualitative content analysis [34]. However, the large amount of data may, as well as the authors professional backgrounds, have influenced the results and negatively affected consistency [34]. Consequently, to ensure that the results were grounded in data, preliminary results were discussed with external auditors, and study participants were offered the opportunity to read and provide feedback, in keeping with Yin [29]. Efforts were made to ensure transparency throughout the research process, for example, by including citations in the results section. However, the exact professional source could not be disclosed as this could risk the identification of specific persons.

## Key learnings and conclusions

This study indicated the FACT model includes several critical components essential for supporting service users’ participation in everyday life and personal recovery. The multiprofessional team, working in a structured and integrated manner with Social Services while bridging gaps between other networks, was key to adapting a flexible and continuous approach to care and support. This in turn, fostered relationships between service users and team members while emphasising service users’ strengths and resources in their everyday lives, thereby promoting empowerment and reducing stigma. The study results add to the existing knowledge of the FACT model, by clarifying how its service delivery perspective influences service users’ participation in everyday life and personal recovery. However, further research using different study designs is needed to complement this knowledge, including RCTs to assess the effectiveness of FACT.
